# Supplementary material for: Iterative improvement in the automatic modular design of robot swarms
Source: PeerJ Comput Sci. 2020 Dec 7;6:e322. doi: 10.7717/peerj-cs.322 (PMC7924708; doi:10.7717/peerj-cs.322)
Supplement: Supplemental Information 3 [file peerj-cs-06-322-s003.zip › argos3/doc/api/standalone/a00622_source.html]

ARGoS: plugins/simulator/physics\_engines/pointmass3d/pointmass3d\_cylinder\_model.h Source File


- Main Page
- Related Pages
- Namespaces
- Classes
- Files

- File List
- File Members

# plugins/simulator/physics\_engines/pointmass3d/pointmass3d\_cylinder\_model.h

Go to the documentation of this file.

```
00001 
00007 #ifndef POINTMASS3D_CYLINDER_H
00008 #define POINTMASS3D_CYLINDER_H
00009 
00010 namespace argos {
00011    class CPointMass3DEngine;
00012    class CPointMass3DCylinderModel;
00013    class CQuadRotorEntity;
00014 }
00015 
00016 #include <argos3/plugins/simulator/physics_engines/pointmass3d/pointmass3d_model.h>
00017 #include <argos3/plugins/simulator/entities/quadrotor_entity.h>
00018 #include <argos3/plugins/simulator/entities/cylinder_entity.h>
00019 
00020 namespace argos {
00021 
00022    class CPointMass3DCylinderModel : public CPointMass3DModel {
00023 
00024    public:
00025       
00026       CPointMass3DCylinderModel(CPointMass3DEngine& c_engine,
00027                                 CCylinderEntity& c_cylinder);
00028       virtual ~CPointMass3DCylinderModel() {}
00029       
00030       virtual void UpdateFromEntityStatus() {}
00031       virtual void Step() {}
00032 
00033       virtual void CalculateBoundingBox();
00034 
00035       virtual bool CheckIntersectionWithRay(Real& f_t_on_ray,
00036                                             const CRay3& c_ray) const;
00037 
00038    private:
00039 
00040       CCylinderEntity& m_cCylinderEntity;
00041    };
00042 
00043 }
00044 
00045 #endif
```

---

Generated on 10 Jul 2018 for ARGoS by 
 1.6.1
